# Tetra­kis-μ-l-alanine-κ^8^ *O*:*O*′-bis­[tetra­aqua­terbium(III)] hexa­perchlorate

**DOI:** 10.1107/S1600536810002448

**Published:** 2010-01-23

**Authors:** Musa E. Mohamed, Deepak Chopra, K. N. Venugopal, Thavendran Govender, Hendrik G. Kruger, Glenn E. M. Maguire

**Affiliations:** a School of Chemistry, University of KwaZulu-Natal, Durban 4000, South Africa; b Department of Chemistry, Indian Institute of Science Education and Research, Bhopal 462 023, India; c Department of Pharmaceutical Chemistry, Al-Ameen College of Pharmacy, Bangalore 560 027, Karnataka, India; d School of Pharmacy and Pharmacology, University of KwaZulu-Natal, Durban 4000, South Africa

## Abstract

The asymmetric unit of the title compound, [Tb_2_(C_3_H_7_NO_2_)_4_(H_2_O)_8_](ClO_4_)_6_, contains a dinuclear cation and six perchlorate anions, one of which is disordered. In the cation, the four l-alanine mol­ecules are present in their zwitterionic form and bridge two Tb^3+^ ions through their carboxyl­ate O atoms. Each Tb atom is also coordinated by four water mol­ecules in a square-anti­prismatic geometry. In the crystal structure, the cations and anions are held together *via* inter­molecular O—H⋯O and N—H⋯O hydrogen bonds.

## Related literature

For applications of terbium complexes, see: Ropp (2004 ▶). For complexes of rare-earth ions, see: Ngoan *et al.* (1988 ▶); Glowiak *et al.* (1991 ▶, 1996 ▶); Hu *et al.* (1995 ▶); Tianzhu *et al.* (1987 ▶).
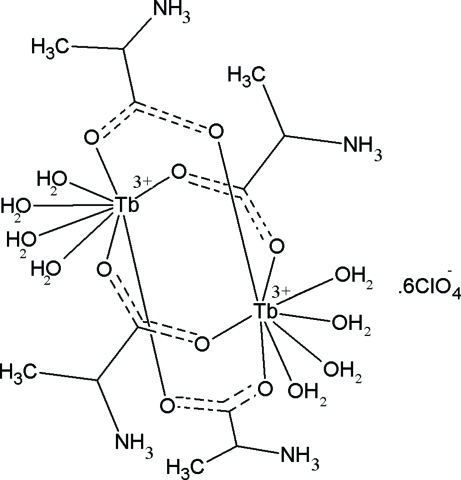


## Experimental

### 

#### Crystal data

[Tb_2_(C_3_H_7_NO_2_)_4_(H_2_O)_8_](ClO_4_)_6_
*M* *_r_* = 1415.05
Triclinic, 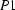

*a* = 10.7703 (3) Å
*b* = 10.7766 (2) Å
*c* = 11.3521 (3) Å
α = 79.345 (2)°
β = 65.390 (3)°
γ = 67.658 (2)°
*V* = 1107.44 (5) Å^3^
*Z* = 1
Mo *K*α radiation
μ = 3.65 mm^−1^
*T* = 100 K
0.40 × 0.40 × 0.40 mm

#### Data collection

Oxford Diffraction Excalibur2 CCD diffractometer
Absorption correction: multi-scan (Blessing, 1995 ▶, 1997 ▶) *T*_min_ = 0.637, *T*_max_ = 0.780
11115 measured reflections
8505 independent reflections
8128 reflections with *I* > 2σ(*I*)
*R*_int_ = 0.027

#### Refinement

*R*[*F* ^2^ > 2σ(*F* ^2^)] = 0.034
*wR*(*F* ^2^) = 0.107
*S* = 1.09
8505 reflections
639 parameters
47 restraints
H atoms treated by a mixture of independent and constrained refinement
Δρ_max_ = 1.42 e Å^−3^
Δρ_min_ = −2.57 e Å^−3^
Absolute structure: Flack (1983 ▶), 770 Friedel pairs
Flack parameter: 0.006 (9)

### 

Data collection: *CrysAlis CCD* (Oxford Diffraction, 2003 ▶); cell refinement: *CrysAlis CCD*; data reduction: *CrysAlis RED* (Oxford Diffraction, 2003 ▶); program(s) used to solve structure: *SHELXS97* (Sheldrick, 2008 ▶); program(s) used to refine structure: *SHELXL97* (Sheldrick, 2008 ▶); molecular graphics: *ORTEP-3 for Windows* (Farrugia, 1997 ▶); software used to prepare material for publication: *PLATON* (Spek, 2009 ▶).

## Supplementary Material

Crystal structure: contains datablocks global, I. DOI: 10.1107/S1600536810002448/cv2689sup1.cif

Structure factors: contains datablocks I. DOI: 10.1107/S1600536810002448/cv2689Isup2.hkl

Additional supplementary materials:  crystallographic information; 3D view; checkCIF report

## Figures and Tables

**Table 1:** Hydrogen-bond geometry (Å, °)

*D*—H⋯*A*	*D*—H	H⋯*A*	*D*⋯*A*	*D*—H⋯*A*
N2—H23⋯O12*S*	0.91	2.20	2.928 (15)	136
N3—H31⋯O17*S*	0.91	2.32	3.032 (15)	136
O14—H14*B*⋯O25*S*	0.85 (10)	2.01 (9)	2.754 (15)	145 (6)
O5—H5*A*⋯O3*S*^i^	0.86 (4)	2.00 (4)	2.809 (10)	156 (3)
O4—H4*B*⋯O9*S*^i^	0.85 (5)	2.37 (6)	3.05 (2)	137 (3)
N2—H22⋯O4*S*^ii^	0.91	2.23	3.022 (12)	145
N2—H21⋯O15*S*^ii^	0.91	2.11	2.768 (19)	129
N1—H13⋯O16*S*^ii^	0.91	2.02	2.906 (13)	163
N2—H22⋯O2*S*^ii^	0.91	2.22	3.016 (12)	147
N4—H42⋯O7*SB*^iii^	0.91	2.10	2.98 (5)	163
N3—H33⋯O22*S*^iii^	0.91	1.94	2.822 (19)	164
N4—H41⋯O24*S*^iii^	0.91	2.18	3.033 (12)	156
N4—H42⋯O5*S*^iii^	0.91	2.31	3.049 (13)	139
N1—H11⋯O6*S*^iv^	0.91	2.20	3.002 (15)	147
O3—H3*B*⋯O6*S*^iv^	0.85 (5)	2.33 (7)	3.149 (15)	161 (4)
N1—H12⋯O4*S*^v^	0.91	2.09	2.981 (11)	165
N2—H21⋯O23*S*^vi^	0.91	2.30	2.924 (10)	125
O3—H3*A*⋯O23*S*^vi^	0.85 (6)	2.04 (5)	2.882 (10)	171 (4)
O4—H4*A*⋯O20*S*^vi^	0.86 (6)	2.01 (4)	2.826 (10)	158 (5)
N4—H43⋯O19*S*^vii^	0.91	2.16	3.017 (11)	156
O16—H16*B*⋯O20*S*^vii^	0.84 (10)	2.16 (5)	2.794 (11)	133 (4)
N3—H32⋯O5*S*^viii^	0.91	2.06	2.926 (12)	159
O13—H13*A*⋯O8^ix^	0.86 (3)	2.01 (3)	2.863 (10)	174 (5)

Symmetry codes: (i) 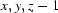; (ii) 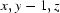; (iii) 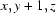; (iv) 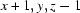; (v) 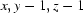; (vi) 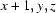; (vii) 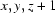; (viii) 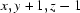; (ix) 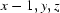.

**Fig. 1.**
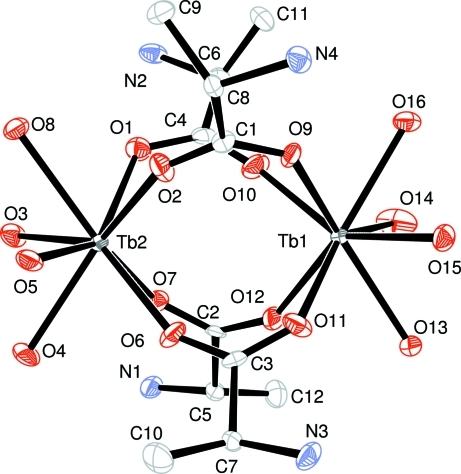
View of the cation in (I) showing the atomic numbering and 50% probability displacement ellipsoids. H atoms have been omitted for clarity.

## supplementary crystallographic information

### Comment

Structural determinations of complexes of rare-earth metals with amino acids are of interest to understand the coordination chemistry of these important class of compounds and to utilize in different optical devices (Ropp, 2004).

In this regard, different complexes, with DL-alanine as the amino acid, containing chloride ions as the counter-ion with the rare-earth metal ion being holmium (Ngoan *et al.*, 1988) and dysprosium (Glowiak *et al.*, 1991) have been synthesized and characterized structurally. The commonly observed inorganic counterions are either perchlorate or chloride anions. It has been observed that depending on the counterion present, the crystal structure contains motifs forming either dimers, chains or network structure in the crystal lattice (Hu *et al.*, 1995, and references therein). Keeping in mind the structural diversity associated with these complexes, we report here the structure of a terbium complex with L-alanine, (I), as extension of the already determined crystal structures.

The title compound (I) crystallizes in the triclinic non-centrosymmetric space group P1. Analogous complexes of neodymium (existing as dimorphs; Glowiak *et al.*, 1996), yttrium (Tianzhu *et al.*, 1987),and erbium (Hu *et al.*, 1995) have also been characterized structurally. The present complex is isostructural with the triclinic form of the neodynium complex which also crystallizes in the triclinic space group P1. The dimeric structure of the complex is depicted in Fig.1. The terbium atom exists in a distorted square-antiprism geometry, having a coordination number of eight. The complex contains two eight-membered rings in the dinuclear cluster, the dihedral angles between these being 88.1 (1)°.

The crystal structure is composed of discrete dinuclear clusters of terbium metal atoms bridged by the carboxyl group of the L-alanine ligand. The ligand exists in the zwitterionic form. The Tb–O(carboxyl) distances lie in the range of 2.274 (6)-2.376 (6)Å while those of Tb–O(water) between 2.358 (8)Å and 2.539 (6)Å. The Tb—Tb distance is 4.367 (3)Å. The dinuclear cations are separated by perchlorate ions, which form hydrogen bonds between coordinated water molecules and the amino groups (Table 1).

### Experimental

An aqueous solution of terbium perchlorate was prepared by digesting (0.15 gm) terbium oxide in concentrated perchloric acid (2 ml), a suitable concentration of terbium perchlorate (0.33 g, 2 mmol) was achieved by diluting the concentrated solution with 4 ml distilled water. L-alanine (0.10 g, 1 mmol) was added as solid to the above aqueous solution of terbium perchlorate. The mixture was stirred at about 80C on a heating plate while an aqueous solution of NaOH (0.5M) was added dropwise to cause an incipient but permanent precipitate, pH=4. The mixture was then filtered, and the filtrate was then reduced to about 4 ml. The hot solution was tightly covered and allowed to evaporate gradually at room temperature. The crystalline precipitate appeared in about 7 days. The solid was collected by filtration, washed with cold diethyl ether/THF 1:1 v/v, and dried under vacuum in a desiccator charged with silica gel. The melting point is 241C. The presence of terbium metal was detected by xylenol orange indicator.

### Refinement

All the amino, methine and methyl hydrogen atoms were positioned geometrically and refined using a riding model with d(N—H) = 0.91Å, U_iso_(H) = 1.2U_eq_ (N) and d(C—H) = 0.96Å and 0.98Å, U_iso_(H)=1.5U_eq_(C).

All the hydrogen atoms of the water molecule coordinated to the metal ion, were refined using geometrical bond restraints, the d(O—H) = 0.85 (5)Å and d(H···H) = 1.37 (2) Å, respectively.

The number of perchlorate ions present in the asymmetric unit is six, out of which one is disordered, the occupancies of the disoredred oxygen atom refined to 0.71 (10) and 0.29 (10), respectively. The Cl—O bond distances lie in the range of acceptable bond lengths, between 1.392 (10)-1.52 (5)Å.

### Crystal data

**Table:**

[Tb_2_(C_3_H_7_NO_2_)_4_(H_2_O)_8_](ClO_4_)_6_	*Z* = 1
*M**_r_* = 1415.05	*F*(000) = 696
Triclinic, *P*1	*D*_x_ = 2.122 Mg m^−^^3^
Hall symbol: P 1	Mo *K*α radiation, λ = 0.71073 Å
*a* = 10.7703 (3) Å	Cell parameters from 665 reflections
*b* = 10.7766 (2) Å	θ = 1.7–25.9°
*c* = 11.3521 (3) Å	µ = 3.65 mm^−^^1^
α = 79.345 (2)°	*T* = 100 K
β = 65.390 (3)°	Block, colourless
γ = 67.658 (2)°	0.40 × 0.40 × 0.40 mm
*V* = 1107.44 (5) Å^3^	

### Data collection

**Table:**

Oxford Diffraction Excalibur2 CCD diffractometer	8505 independent reflections
Radiation source: fine-focus sealed tube	8128 reflections with *I* > 2σ(*I*)
graphite	*R*_int_ = 0.027
ω and 2θ scans	θ_max_ = 32.1°, θ_min_ = 2.8°
Absorption correction: multi-scan (Blessing, 1995, 1997)	*h* = −15→15
*T*_min_ = 0.637, *T*_max_ = 0.780	*k* = −11→15
11115 measured reflections	*l* = −16→16

### Refinement

**Table:**

Refinement on *F*^2^	Secondary atom site location: difference Fourier map
Least-squares matrix: full	Hydrogen site location: inferred from neighbouring sites
*R*[*F*^2^ > 2σ(*F*^2^)] = 0.034	H atoms treated by a mixture of independent and constrained refinement
*wR*(*F*^2^) = 0.107	*w* = 1/[σ^2^(*F*_o_^2^) + (0.0814*P*)^2^ + 0.8066*P*] where *P* = (*F*_o_^2^ + 2*F*_c_^2^)/3
*S* = 1.09	(Δ/σ)_max_ < 0.001
8505 reflections	Δρ_max_ = 1.42 e Å^−^^3^
639 parameters	Δρ_min_ = −2.57 e Å^−^^3^
47 restraints	Absolute structure: Flack (1983), 770 Friedel pairs
Primary atom site location: structure-invariant direct methods	Flack parameter: 0.006 (9)

### Special details

**Table:**

Geometry. All esds (except the esd in the dihedral angle between two l.s. planes) are estimated using the full covariance matrix. The cell esds are taken into account individually in the estimation of esds in distances, angles and torsion angles; correlations between esds in cell parameters are only used when they are defined by crystal symmetry. An approximate (isotropic) treatment of cell esds is used for estimating esds involving l.s. planes.
Refinement. Refinement of F^2^ against ALL reflections. The weighted R-factor wR and goodness of fit S are based on F^2^, conventional R-factors R are based on F, with F set to zero for negative F^2^. The threshold expression of F^2^ > 2sigma(F^2^) is used only for calculating R-factors(gt) etc. and is not relevant to the choice of reflections for refinement. R-factors based on F^2^ are statistically about twice as large as those based on F, and R- factors based on ALL data will be even larger.

### Fractional atomic coordinates and isotropic or equivalent isotropic displacement parameters (Å^2^)

**Table:**

	*x*	*y*	*z*	*U*_iso_*/*U*_eq_	Occ. (<1)
C1	0.4190 (9)	0.6940 (9)	0.1555 (8)	0.0145 (14)	
C2	0.5789 (9)	0.3191 (9)	−0.1630 (7)	0.0140 (14)	
C3	0.4097 (9)	0.6629 (9)	−0.1672 (8)	0.0150 (14)	
C4	0.6005 (9)	0.3273 (9)	0.1564 (8)	0.0168 (15)	
C5	0.6141 (7)	0.2316 (8)	−0.2732 (7)	0.0170 (12)	
H5	0.5923	0.2941	−0.3445	0.020*	
C6	0.6109 (8)	0.2245 (8)	0.2672 (7)	0.0193 (14)	
H6	0.5886	0.1472	0.2543	0.023*	
C7	0.3826 (7)	0.7421 (8)	−0.2844 (6)	0.0158 (12)	
H7	0.4093	0.6759	−0.3495	0.019*	
C8	0.3880 (7)	0.8167 (8)	0.2267 (7)	0.0158 (11)	
H8	0.4013	0.8918	0.1616	0.019*	
N1	0.7733 (8)	0.1558 (9)	−0.3250 (8)	0.0223 (16)	
H11	0.8229	0.2139	−0.3459	0.033*	
H12	0.7976	0.1121	−0.3970	0.033*	
H13	0.7966	0.0950	−0.2637	0.033*	
N2	0.7634 (9)	0.1745 (9)	0.2602 (8)	0.0240 (15)	
H21	0.8252	0.1403	0.1808	0.036*	
H22	0.7730	0.1093	0.3225	0.036*	
H23	0.7850	0.2436	0.2730	0.036*	
C9	0.4869 (8)	0.7915 (9)	0.2993 (7)	0.0246 (14)	
H9A	0.4692	0.7234	0.3684	0.037*	
H9C	0.4666	0.8751	0.3372	0.037*	
H9B	0.5884	0.7599	0.2390	0.037*	
C10	0.4726 (9)	0.8325 (9)	−0.3490 (8)	0.0291 (16)	
H10A	0.4406	0.9054	−0.2913	0.044*	
H10B	0.4602	0.8704	−0.4304	0.044*	
H10C	0.5751	0.7803	−0.3671	0.044*	
N3	0.2240 (8)	0.8194 (9)	−0.2458 (8)	0.0215 (14)	
H31	0.1727	0.7626	−0.2074	0.032*	
H32	0.2055	0.8596	−0.3175	0.032*	
H33	0.1973	0.8832	−0.1891	0.032*	
N4	0.2318 (8)	0.8572 (8)	0.3187 (7)	0.0176 (13)	
H41	0.1740	0.8718	0.2740	0.026*	
H42	0.2082	0.9337	0.3582	0.026*	
H43	0.2185	0.7905	0.3797	0.026*	
C11	0.5048 (9)	0.2818 (10)	0.3982 (7)	0.0303 (17)	
H11A	0.4054	0.3085	0.4023	0.045*	
H11B	0.5222	0.3604	0.4107	0.045*	
H11C	0.5179	0.2137	0.4663	0.045*	
C12	0.5248 (8)	0.1422 (8)	−0.2365 (8)	0.0259 (14)	
H12A	0.4220	0.1973	−0.2119	0.039*	
H12B	0.5390	0.0829	−0.1631	0.039*	
H12C	0.5551	0.0880	−0.3105	0.039*	
O1	0.7135 (7)	0.3573 (7)	0.0837 (6)	0.0216 (12)	
Cl2S	0.8570 (2)	0.8811 (2)	0.4648 (2)	0.0180 (4)	
O2	0.5450 (7)	0.6491 (7)	0.0736 (6)	0.0228 (13)	
O2S	0.7578 (8)	0.9092 (8)	0.4010 (7)	0.0311 (14)	
Cl5S	0.1466 (2)	0.0542 (2)	0.0312 (2)	0.0203 (4)	
O5S	0.1243 (8)	0.0075 (8)	0.5644 (8)	0.0317 (15)	
O6	0.5399 (6)	0.6135 (7)	−0.1774 (6)	0.0229 (12)	
O6S	0.0345 (10)	0.2428 (10)	0.5709 (11)	0.045 (3)	
O7	0.6865 (6)	0.3358 (7)	−0.1563 (6)	0.0198 (11)	
O3	0.9611 (7)	0.3441 (8)	−0.1569 (6)	0.0239 (14)	
H3A	0.992 (7)	0.293 (7)	−0.101 (4)	0.029*	
H3B	1.002 (7)	0.310 (8)	−0.231 (4)	0.029*	
O4	0.8468 (7)	0.5258 (8)	−0.3247 (6)	0.0254 (14)	
H4A	0.940 (2)	0.494 (10)	−0.364 (4)	0.030*	
H4B	0.808 (4)	0.550 (11)	−0.381 (3)	0.030*	
O5	0.7415 (7)	0.7271 (7)	−0.1561 (6)	0.0208 (12)	
H5A	0.780 (10)	0.754 (5)	−0.235 (2)	0.025*	
H5B	0.712 (10)	0.790 (4)	−0.106 (4)	0.025*	
O8	0.8528 (7)	0.5495 (7)	0.0278 (6)	0.0198 (12)	
H8A	0.853 (10)	0.629 (4)	0.016 (7)	0.024*	
H8B	0.817 (9)	0.529 (8)	0.108 (3)	0.024*	
O13	0.1579 (7)	0.4609 (8)	−0.0296 (6)	0.0237 (14)	
H13A	0.065 (2)	0.488 (10)	−0.007 (5)	0.028*	
H13B	0.196 (5)	0.446 (11)	−0.110 (3)	0.028*	
O14	0.2390 (11)	0.2843 (8)	0.1615 (8)	0.0385 (19)	
H14A	0.277 (12)	0.234 (6)	0.214 (9)	0.046*	
H14B	0.243 (13)	0.241 (6)	0.104 (6)	0.046*	
O15	0.0449 (6)	0.6566 (7)	0.1651 (6)	0.0201 (12)	
H15A	0.028 (5)	0.740 (2)	0.152 (9)	0.024*	
H15B	−0.034 (3)	0.641 (4)	0.196 (9)	0.024*	
O16	0.1667 (7)	0.4676 (8)	0.3269 (6)	0.0219 (13)	
H16A	0.085 (6)	0.456 (11)	0.359 (5)	0.026*	
H16B	0.181 (8)	0.506 (9)	0.375 (4)	0.026*	
O9	0.3161 (6)	0.6520 (7)	0.1832 (6)	0.0193 (12)	
O20S	0.1478 (6)	0.4809 (7)	−0.4224 (6)	0.0237 (12)	
O10	0.4811 (7)	0.3768 (7)	0.1443 (6)	0.0263 (13)	
Cl3S	0.7685 (2)	0.4507 (2)	0.41047 (19)	0.0217 (4)	
O11	0.3020 (7)	0.6551 (7)	−0.0709 (6)	0.0230 (13)	
O12	0.4490 (7)	0.3679 (7)	−0.0932 (6)	0.0214 (12)	
O9S	0.8749 (14)	0.5005 (13)	0.4016 (16)	0.090 (5)	
Cl6S	0.8241 (2)	0.9367 (2)	−0.0224 (2)	0.0245 (4)	
O13S	0.6832 (9)	0.9455 (9)	−0.0124 (10)	0.048 (2)	
O14S	0.9066 (9)	0.7981 (7)	−0.0050 (9)	0.0415 (17)	
O17S	0.0746 (7)	0.6455 (8)	−0.2755 (7)	0.0395 (16)	
O18S	0.2996 (7)	0.4729 (8)	−0.3200 (6)	0.0281 (13)	
O15S	0.8045 (12)	1.0152 (12)	0.0727 (13)	0.064 (4)	
O23S	0.0356 (7)	0.1853 (7)	0.0532 (7)	0.0356 (14)	
O24S	0.0912 (7)	−0.0414 (7)	0.1209 (6)	0.0329 (13)	
O25S	0.2664 (8)	0.0614 (8)	0.0544 (9)	0.0421 (18)	
O12S	0.8133 (8)	0.3067 (7)	0.4314 (7)	0.0385 (15)	
O10S	0.7326 (11)	0.4879 (9)	0.2992 (7)	0.045 (2)	
Cl1S	0.1624 (2)	0.1275 (2)	0.5306 (2)	0.0220 (4)	
O7SA	0.255 (5)	0.1305 (19)	0.3977 (15)	0.057 (11)	0.71 (10)
O7SB	0.181 (10)	0.130 (3)	0.390 (3)	0.037 (17)	0.29 (10)
O16S	0.9019 (9)	0.9741 (11)	−0.1509 (8)	0.066 (3)	
O19S	0.2652 (8)	0.6408 (9)	−0.4749 (7)	0.0414 (16)	
O8S	0.2530 (13)	0.1202 (11)	0.5942 (15)	0.072 (4)	
O22S	0.1939 (15)	0.0213 (13)	−0.0991 (10)	0.060 (3)	
O26S	0.6419 (13)	0.5062 (8)	0.5196 (8)	0.078 (4)	
Tb1	0.28195 (2)	0.48987 (2)	0.094542 (19)	0.01289 (8)	
Tb2	0.719556 (19)	0.510671 (19)	−0.093565 (17)	0.01263 (8)	
Cl4S	0.1964 (2)	0.5602 (2)	−0.37293 (18)	0.0218 (4)	
O1S	0.9780 (8)	0.7615 (8)	0.4134 (7)	0.0265 (15)	
O3S	0.7844 (8)	0.8652 (9)	0.6027 (7)	0.0308 (16)	
O4S	0.9085 (7)	0.9956 (7)	0.4380 (6)	0.0219 (12)	

### Atomic displacement parameters (Å^2^)

**Table:**

	*U*^11^	*U*^22^	*U*^33^	*U*^12^	*U*^13^	*U*^23^
C1	0.018 (3)	0.011 (3)	0.017 (3)	−0.005 (3)	−0.008 (3)	−0.002 (3)
C2	0.019 (3)	0.019 (4)	0.005 (3)	−0.008 (3)	−0.004 (2)	0.001 (3)
C3	0.020 (3)	0.015 (3)	0.015 (3)	−0.005 (3)	−0.012 (3)	−0.002 (3)
C4	0.019 (3)	0.017 (3)	0.013 (3)	−0.001 (3)	−0.007 (2)	−0.006 (3)
C5	0.015 (3)	0.022 (3)	0.012 (3)	−0.005 (2)	−0.003 (2)	−0.005 (2)
C6	0.019 (3)	0.020 (3)	0.018 (3)	−0.006 (3)	−0.009 (2)	0.005 (3)
C7	0.013 (3)	0.021 (3)	0.013 (3)	−0.006 (2)	−0.005 (2)	0.004 (2)
C8	0.012 (3)	0.021 (3)	0.013 (3)	−0.007 (2)	−0.001 (2)	−0.003 (2)
N1	0.018 (3)	0.029 (4)	0.020 (3)	−0.005 (3)	−0.006 (3)	−0.011 (3)
N2	0.026 (4)	0.025 (4)	0.016 (3)	−0.002 (3)	−0.011 (3)	0.001 (3)
C9	0.022 (3)	0.035 (4)	0.021 (3)	−0.010 (3)	−0.010 (2)	−0.007 (3)
C10	0.030 (4)	0.029 (4)	0.026 (3)	−0.013 (3)	−0.011 (3)	0.012 (3)
N3	0.014 (3)	0.024 (3)	0.027 (3)	−0.001 (3)	−0.012 (2)	−0.003 (3)
N4	0.015 (3)	0.018 (3)	0.018 (3)	−0.002 (3)	−0.005 (2)	−0.002 (3)
C11	0.022 (3)	0.046 (5)	0.016 (3)	−0.012 (3)	−0.002 (3)	0.000 (3)
C12	0.023 (3)	0.026 (3)	0.028 (3)	−0.011 (3)	−0.005 (3)	−0.005 (3)
O1	0.024 (3)	0.021 (3)	0.020 (3)	−0.008 (2)	−0.009 (2)	0.001 (2)
Cl2S	0.0172 (8)	0.0215 (9)	0.0129 (7)	−0.0069 (7)	−0.0037 (6)	0.0006 (7)
O2	0.017 (3)	0.024 (3)	0.019 (3)	−0.005 (2)	−0.0014 (19)	0.000 (2)
O2S	0.033 (3)	0.037 (3)	0.036 (3)	−0.019 (3)	−0.023 (3)	0.010 (3)
Cl5S	0.0198 (8)	0.0230 (8)	0.0198 (7)	−0.0055 (6)	−0.0099 (6)	−0.0024 (6)
O5S	0.041 (4)	0.026 (3)	0.037 (3)	−0.016 (3)	−0.020 (3)	−0.001 (3)
O6	0.021 (3)	0.025 (3)	0.029 (3)	−0.008 (2)	−0.018 (2)	0.003 (2)
O6S	0.026 (4)	0.029 (4)	0.078 (7)	−0.001 (3)	−0.019 (4)	−0.017 (4)
O7	0.019 (2)	0.024 (3)	0.019 (2)	−0.005 (2)	−0.0081 (19)	−0.006 (2)
O3	0.016 (3)	0.029 (4)	0.017 (3)	0.000 (3)	−0.003 (2)	−0.004 (3)
O4	0.020 (3)	0.036 (4)	0.011 (2)	−0.006 (3)	0.001 (2)	−0.006 (2)
O5	0.028 (3)	0.026 (3)	0.012 (2)	−0.015 (2)	−0.007 (2)	0.004 (2)
O8	0.019 (3)	0.024 (3)	0.020 (3)	−0.006 (2)	−0.011 (2)	−0.003 (2)
O13	0.017 (3)	0.041 (4)	0.015 (3)	−0.010 (3)	−0.006 (2)	−0.004 (3)
O14	0.070 (6)	0.028 (4)	0.030 (4)	−0.028 (4)	−0.022 (4)	0.004 (3)
O15	0.014 (3)	0.021 (3)	0.023 (3)	−0.003 (2)	−0.008 (2)	0.000 (2)
O16	0.026 (3)	0.025 (3)	0.015 (3)	−0.008 (3)	−0.010 (2)	0.001 (2)
O9	0.013 (2)	0.027 (3)	0.021 (3)	−0.008 (2)	−0.0069 (19)	−0.006 (2)
O20S	0.023 (3)	0.034 (3)	0.020 (2)	−0.010 (2)	−0.0084 (19)	−0.011 (2)
O10	0.021 (3)	0.031 (3)	0.028 (3)	−0.001 (2)	−0.016 (2)	−0.003 (2)
Cl3S	0.0247 (9)	0.0269 (9)	0.0205 (9)	−0.0134 (7)	−0.0129 (7)	0.0034 (7)
O11	0.023 (3)	0.024 (3)	0.013 (2)	−0.003 (2)	−0.004 (2)	0.003 (2)
O12	0.017 (2)	0.028 (3)	0.020 (3)	−0.007 (2)	−0.006 (2)	−0.007 (2)
O9S	0.085 (8)	0.076 (8)	0.163 (13)	−0.062 (7)	−0.094 (9)	0.064 (9)
Cl6S	0.0279 (10)	0.0223 (9)	0.0229 (8)	−0.0021 (7)	−0.0127 (7)	−0.0064 (7)
O13S	0.045 (5)	0.039 (4)	0.071 (6)	−0.004 (4)	−0.035 (4)	−0.014 (4)
O14S	0.054 (5)	0.024 (3)	0.063 (5)	−0.011 (3)	−0.043 (4)	0.007 (3)
O17S	0.019 (3)	0.047 (4)	0.050 (4)	−0.005 (3)	−0.007 (2)	−0.026 (3)
O18S	0.030 (3)	0.040 (4)	0.020 (3)	−0.012 (3)	−0.015 (2)	0.000 (2)
O15S	0.054 (6)	0.067 (7)	0.088 (9)	0.000 (5)	−0.039 (6)	−0.054 (7)
O23S	0.030 (3)	0.028 (3)	0.034 (3)	0.004 (3)	−0.011 (3)	−0.004 (3)
O24S	0.036 (3)	0.033 (3)	0.034 (3)	−0.017 (3)	−0.017 (3)	0.009 (3)
O25S	0.029 (4)	0.035 (4)	0.068 (5)	−0.004 (3)	−0.027 (4)	−0.010 (4)
O12S	0.041 (4)	0.028 (3)	0.036 (3)	−0.005 (3)	−0.014 (3)	0.005 (3)
O10S	0.079 (6)	0.040 (4)	0.024 (3)	−0.023 (4)	−0.026 (4)	0.002 (3)
Cl1S	0.0220 (9)	0.0201 (9)	0.0185 (8)	−0.0027 (7)	−0.0064 (7)	−0.0012 (7)
O7SA	0.08 (2)	0.034 (6)	0.014 (5)	−0.010 (8)	0.006 (7)	0.007 (4)
O7SB	0.07 (3)	0.014 (10)	0.009 (9)	−0.015 (14)	0.005 (12)	0.003 (7)
O16S	0.042 (4)	0.068 (6)	0.041 (4)	0.009 (4)	−0.005 (3)	0.020 (4)
O19S	0.039 (4)	0.053 (4)	0.035 (3)	−0.029 (3)	−0.014 (3)	0.023 (3)
O8S	0.074 (7)	0.051 (6)	0.136 (10)	−0.032 (5)	−0.084 (8)	0.023 (6)
O22S	0.105 (9)	0.054 (6)	0.023 (3)	−0.035 (6)	−0.018 (4)	−0.007 (3)
O26S	0.105 (8)	0.028 (4)	0.042 (4)	−0.009 (5)	0.021 (5)	−0.011 (3)
Tb1	0.01223 (14)	0.01682 (17)	0.01058 (14)	−0.00513 (12)	−0.00486 (11)	−0.00088 (12)
Tb2	0.01099 (14)	0.01639 (16)	0.01099 (14)	−0.00412 (12)	−0.00485 (11)	−0.00105 (11)
Cl4S	0.0213 (8)	0.0299 (9)	0.0168 (8)	−0.0127 (7)	−0.0065 (6)	0.0006 (7)
O1S	0.023 (3)	0.025 (4)	0.027 (3)	−0.005 (3)	−0.004 (2)	−0.011 (3)
O3S	0.029 (4)	0.033 (4)	0.019 (3)	−0.009 (3)	−0.001 (3)	0.002 (3)
O4S	0.028 (3)	0.022 (3)	0.020 (2)	−0.012 (2)	−0.009 (2)	−0.001 (2)

### Geometric parameters (Å, °)

**Table:**

C1—O2	1.247 (10)	O2—Tb2	2.304 (7)
C1—O9	1.254 (10)	Cl5S—O22S	1.415 (10)
C1—C8	1.533 (11)	Cl5S—O24S	1.424 (6)
C2—O12	1.238 (10)	Cl5S—O23S	1.444 (7)
C2—O7	1.272 (10)	Cl5S—O25S	1.451 (7)
C2—C5	1.539 (10)	O5S—Cl1S	1.442 (8)
C3—O11	1.238 (10)	O6—Tb2	2.322 (6)
C3—O6	1.258 (10)	O6S—Cl1S	1.426 (9)
C3—C7	1.516 (10)	O7—Tb2	2.324 (6)
C4—O10	1.247 (10)	O3—Tb2	2.424 (7)
C4—O1	1.278 (11)	O3—H3A	0.85 (6)
C4—C6	1.525 (12)	O3—H3B	0.85 (5)
C5—N1	1.492 (10)	O4—Tb2	2.410 (6)
C5—C12	1.500 (11)	O4—H4A	0.86 (6)
C5—H5	1.0000	O4—H4B	0.85 (6)
C6—N2	1.493 (11)	O5—Tb2	2.380 (7)
C6—C11	1.512 (11)	O5—H5A	0.86 (2)
C6—H6	1.0000	O5—H5B	0.84 (5)
C7—N3	1.495 (10)	O8—Tb2	2.539 (6)
C7—C10	1.513 (11)	O8—H8A	0.84 (6)
C7—H7	1.0000	O8—H8B	0.85 (2)
C8—N4	1.504 (9)	O13—Tb1	2.432 (6)
C8—C9	1.521 (10)	O13—H13A	0.86 (6)
C8—H8	1.0000	O13—H13B	0.85 (2)
N1—H11	0.9100	O14—Tb1	2.358 (8)
N1—H12	0.9100	O14—H14A	0.86 (11)
N1—H13	0.9100	O14—H14B	0.84 (6)
N2—H21	0.9100	O15—Tb1	2.394 (6)
N2—H22	0.9100	O15—H15A	0.85 (2)
N2—H23	0.9100	O15—H15B	0.84 (6)
C9—H9A	0.9800	O16—Tb1	2.413 (6)
C9—H9C	0.9800	O16—H16A	0.85 (8)
C9—H9B	0.9800	O16—H16B	0.83 (8)
C10—H10A	0.9800	O9—Tb1	2.376 (6)
C10—H10B	0.9800	O20S—Cl4S	1.442 (6)
C10—H10C	0.9800	O10—Tb1	2.274 (6)
N3—H31	0.9100	Cl3S—O9S	1.402 (9)
N3—H32	0.9100	Cl3S—O26S	1.414 (8)
N3—H33	0.9100	Cl3S—O10S	1.417 (8)
N4—H41	0.9100	Cl3S—O12S	1.444 (7)
N4—H42	0.9100	O11—Tb1	2.337 (7)
N4—H43	0.9100	O12—Tb1	2.356 (7)
C11—H11A	0.9800	Cl6S—O15S	1.392 (10)
C11—H11B	0.9800	Cl6S—O16S	1.419 (8)
C11—H11C	0.9800	Cl6S—O13S	1.441 (8)
C12—H12A	0.9800	Cl6S—O14S	1.445 (7)
C12—H12B	0.9800	O17S—Cl4S	1.429 (7)
C12—H12C	0.9800	O18S—Cl4S	1.426 (7)
O1—Tb2	2.352 (7)	Cl1S—O8S	1.410 (9)
Cl2S—O1S	1.438 (8)	Cl1S—O7SA	1.422 (17)
Cl2S—O3S	1.441 (7)	Cl1S—O7SB	1.52 (5)
Cl2S—O2S	1.441 (7)	O19S—Cl4S	1.430 (6)
Cl2S—O4S	1.470 (7)		
			
O2—C1—O9	127.5 (8)	Tb2—O5—H5A	125 (3)
O2—C1—C8	115.2 (7)	Tb2—O5—H5B	126 (3)
O9—C1—C8	117.3 (7)	H5A—O5—H5B	109 (5)
O12—C2—O7	127.5 (7)	Tb2—O8—H8A	111 (4)
O12—C2—C5	116.4 (7)	Tb2—O8—H8B	111 (4)
O7—C2—C5	116.1 (7)	H8A—O8—H8B	112 (5)
O11—C3—O6	126.7 (8)	Tb1—O13—H13A	125 (3)
O11—C3—C7	117.0 (7)	Tb1—O13—H13B	125 (3)
O6—C3—C7	116.3 (7)	H13A—O13—H13B	107 (5)
O10—C4—O1	124.7 (9)	Tb1—O14—H14A	117 (5)
O10—C4—C6	117.3 (8)	Tb1—O14—H14B	117 (5)
O1—C4—C6	118.0 (7)	H14A—O14—H14B	114 (5)
N1—C5—C12	112.6 (7)	Tb1—O15—H15A	125 (3)
N1—C5—C2	108.5 (6)	Tb1—O15—H15B	125 (3)
C12—C5—C2	114.1 (6)	H15A—O15—H15B	110 (5)
N1—C5—H5	107.0	Tb1—O16—H16A	119 (3)
C12—C5—H5	107.0	Tb1—O16—H16B	120 (4)
C2—C5—H5	107.0	H16A—O16—H16B	115 (5)
N2—C6—C11	111.0 (7)	C1—O9—Tb1	134.3 (5)
N2—C6—C4	108.3 (6)	C4—O10—Tb1	170.1 (7)
C11—C6—C4	112.3 (7)	O9S—Cl3S—O26S	108.3 (9)
N2—C6—H6	108.4	O9S—Cl3S—O10S	111.4 (7)
C11—C6—H6	108.4	O26S—Cl3S—O10S	108.1 (7)
C4—C6—H6	108.4	O9S—Cl3S—O12S	111.3 (6)
N3—C7—C10	111.4 (7)	O26S—Cl3S—O12S	106.9 (5)
N3—C7—C3	109.0 (6)	O10S—Cl3S—O12S	110.6 (5)
C10—C7—C3	113.9 (6)	C3—O11—Tb1	129.8 (6)
N3—C7—H7	107.4	C2—O12—Tb1	145.5 (5)
C10—C7—H7	107.4	O15S—Cl6S—O16S	114.4 (8)
C3—C7—H7	107.4	O15S—Cl6S—O13S	108.3 (6)
N4—C8—C9	110.5 (6)	O16S—Cl6S—O13S	108.8 (6)
N4—C8—C1	107.4 (6)	O15S—Cl6S—O14S	110.9 (6)
C9—C8—C1	113.4 (6)	O16S—Cl6S—O14S	105.2 (5)
N4—C8—H8	108.5	O13S—Cl6S—O14S	109.2 (5)
C9—C8—H8	108.5	O8S—Cl1S—O7SA	102 (3)
C1—C8—H8	108.5	O8S—Cl1S—O6S	109.7 (7)
C5—N1—H11	109.5	O7SA—Cl1S—O6S	115.0 (12)
C5—N1—H12	109.5	O8S—Cl1S—O5S	108.2 (6)
H11—N1—H12	109.5	O7SA—Cl1S—O5S	111.3 (16)
C5—N1—H13	109.5	O6S—Cl1S—O5S	110.0 (5)
H11—N1—H13	109.5	O8S—Cl1S—O7SB	135 (4)
H12—N1—H13	109.5	O6S—Cl1S—O7SB	98 (3)
C6—N2—H21	109.5	O5S—Cl1S—O7SB	94 (2)
C6—N2—H22	109.5	O10—Tb1—O11	117.3 (2)
H21—N2—H22	109.5	O10—Tb1—O12	76.9 (2)
C6—N2—H23	109.5	O11—Tb1—O12	76.2 (2)
H21—N2—H23	109.5	O10—Tb1—O14	82.4 (3)
H22—N2—H23	109.5	O11—Tb1—O14	145.0 (3)
C8—C9—H9A	109.5	O12—Tb1—O14	81.3 (3)
C8—C9—H9C	109.5	O10—Tb1—O9	74.7 (2)
H9A—C9—H9C	109.5	O11—Tb1—O9	77.3 (2)
C8—C9—H9B	109.5	O12—Tb1—O9	125.8 (2)
H9A—C9—H9B	109.5	O14—Tb1—O9	137.5 (2)
H9C—C9—H9B	109.5	O10—Tb1—O15	144.7 (2)
C7—C10—H10A	109.5	O11—Tb1—O15	76.9 (2)
C7—C10—H10B	109.5	O12—Tb1—O15	138.1 (2)
H10A—C10—H10B	109.5	O14—Tb1—O15	104.3 (3)
C7—C10—H10C	109.5	O9—Tb1—O15	77.7 (2)
H10A—C10—H10C	109.5	O10—Tb1—O16	80.1 (2)
H10B—C10—H10C	109.5	O11—Tb1—O16	140.5 (2)
C7—N3—H31	109.5	O12—Tb1—O16	143.2 (2)
C7—N3—H32	109.5	O14—Tb1—O16	67.4 (3)
H31—N3—H32	109.5	O9—Tb1—O16	73.7 (2)
C7—N3—H33	109.5	O15—Tb1—O16	71.2 (2)
H31—N3—H33	109.5	O10—Tb1—O13	139.5 (2)
H32—N3—H33	109.5	O11—Tb1—O13	75.4 (2)
C8—N4—H41	109.5	O12—Tb1—O13	68.9 (2)
C8—N4—H42	109.5	O14—Tb1—O13	71.7 (3)
H41—N4—H42	109.5	O9—Tb1—O13	144.0 (2)
C8—N4—H43	109.5	O15—Tb1—O13	73.6 (2)
H41—N4—H43	109.5	O16—Tb1—O13	115.7 (2)
H42—N4—H43	109.5	O2—Tb2—O6	79.9 (2)
C6—C11—H11A	109.5	O2—Tb2—O7	123.2 (2)
C6—C11—H11B	109.5	O6—Tb2—O7	74.8 (2)
H11A—C11—H11B	109.5	O2—Tb2—O1	79.7 (2)
C6—C11—H11C	109.5	O6—Tb2—O1	127.5 (2)
H11A—C11—H11C	109.5	O7—Tb2—O1	77.2 (2)
H11B—C11—H11C	109.5	O2—Tb2—O5	73.9 (2)
C5—C12—H12A	109.5	O6—Tb2—O5	78.4 (2)
C5—C12—H12B	109.5	O7—Tb2—O5	144.1 (2)
H12A—C12—H12B	109.5	O1—Tb2—O5	138.7 (2)
C5—C12—H12C	109.5	O2—Tb2—O4	138.9 (3)
H12A—C12—H12C	109.5	O6—Tb2—O4	74.6 (2)
H12B—C12—H12C	109.5	O7—Tb2—O4	80.3 (2)
C4—O1—Tb2	122.9 (6)	O1—Tb2—O4	141.3 (3)
O1S—Cl2S—O3S	110.6 (5)	O5—Tb2—O4	69.8 (2)
O1S—Cl2S—O2S	109.2 (5)	O2—Tb2—O3	141.8 (2)
O3S—Cl2S—O2S	110.3 (4)	O6—Tb2—O3	138.3 (2)
O1S—Cl2S—O4S	109.7 (4)	O7—Tb2—O3	78.0 (2)
O3S—Cl2S—O4S	109.2 (5)	O1—Tb2—O3	74.6 (2)
O2S—Cl2S—O4S	107.9 (4)	O5—Tb2—O3	108.5 (2)
C1—O2—Tb2	152.4 (6)	O4—Tb2—O3	70.2 (2)
O22S—Cl5S—O24S	112.3 (6)	O2—Tb2—O8	73.8 (2)
O22S—Cl5S—O23S	108.8 (6)	O6—Tb2—O8	144.5 (2)
O24S—Cl5S—O23S	109.6 (4)	O7—Tb2—O8	140.1 (2)
O22S—Cl5S—O25S	109.8 (7)	O1—Tb2—O8	70.7 (2)
O24S—Cl5S—O25S	108.6 (5)	O5—Tb2—O8	71.7 (2)
O23S—Cl5S—O25S	107.6 (5)	O4—Tb2—O8	111.2 (2)
C3—O6—Tb2	153.0 (6)	O3—Tb2—O8	71.3 (2)
C2—O7—Tb2	134.3 (6)	O18S—Cl4S—O17S	110.3 (4)
Tb2—O3—H3A	121 (3)	O18S—Cl4S—O19S	108.3 (4)
Tb2—O3—H3B	120 (3)	O17S—Cl4S—O19S	109.3 (5)
H3A—O3—H3B	113 (5)	O18S—Cl4S—O20S	109.2 (4)
Tb2—O4—H4A	124 (3)	O17S—Cl4S—O20S	109.3 (4)
Tb2—O4—H4B	126 (3)	O19S—Cl4S—O20S	110.5 (4)
H4A—O4—H4B	109 (5)		
			
O12—C2—C5—N1	−166.3 (8)	C2—O12—Tb1—O9	29.2 (12)
O7—C2—C5—N1	15.5 (10)	C2—O12—Tb1—O15	143.4 (10)
O12—C2—C5—C12	−39.8 (11)	C2—O12—Tb1—O16	−83.4 (12)
O7—C2—C5—C12	142.1 (8)	C2—O12—Tb1—O13	171.4 (12)
O10—C4—C6—N2	177.7 (8)	C1—O9—Tb1—O10	56.8 (8)
O1—C4—C6—N2	−3.0 (11)	C1—O9—Tb1—O11	−66.4 (8)
O10—C4—C6—C11	−59.4 (10)	C1—O9—Tb1—O12	−4.1 (9)
O1—C4—C6—C11	120.0 (8)	C1—O9—Tb1—O14	116.6 (8)
O11—C3—C7—N3	12.2 (10)	C1—O9—Tb1—O15	−145.5 (8)
O6—C3—C7—N3	−166.7 (7)	C1—O9—Tb1—O16	140.7 (8)
O11—C3—C7—C10	137.3 (8)	C1—O9—Tb1—O13	−107.9 (8)
O6—C3—C7—C10	−41.6 (10)	C1—O2—Tb2—O6	53.0 (13)
O2—C1—C8—N4	179.1 (7)	C1—O2—Tb2—O7	−11.2 (15)
O9—C1—C8—N4	0.5 (10)	C1—O2—Tb2—O1	−78.4 (14)
O2—C1—C8—C9	−58.6 (10)	C1—O2—Tb2—O5	133.8 (14)
O9—C1—C8—C9	122.9 (8)	C1—O2—Tb2—O4	105.1 (14)
O10—C4—O1—Tb2	−1.7 (13)	C1—O2—Tb2—O3	−126.6 (13)
C6—C4—O1—Tb2	179.0 (5)	C1—O2—Tb2—O8	−151.1 (14)
O9—C1—O2—Tb2	7(2)	C3—O6—Tb2—O2	−36.5 (12)
C8—C1—O2—Tb2	−170.9 (9)	C3—O6—Tb2—O7	92.1 (13)
O11—C3—O6—Tb2	−10.8 (19)	C3—O6—Tb2—O1	32.0 (13)
C7—C3—O6—Tb2	168.0 (9)	C3—O6—Tb2—O5	−112.0 (13)
O12—C2—O7—Tb2	−31.1 (14)	C3—O6—Tb2—O4	176.0 (13)
C5—C2—O7—Tb2	146.8 (6)	C3—O6—Tb2—O3	143.2 (12)
O2—C1—O9—Tb1	−7.2 (15)	C3—O6—Tb2—O8	−78.9 (13)
C8—C1—O9—Tb1	171.1 (5)	C2—O7—Tb2—O2	36.1 (8)
O6—C3—O11—Tb1	−27.6 (13)	C2—O7—Tb2—O6	−30.7 (7)
C7—C3—O11—Tb1	153.6 (6)	C2—O7—Tb2—O1	104.5 (7)
O7—C2—O12—Tb1	−8.9 (18)	C2—O7—Tb2—O5	−73.7 (8)
C5—C2—O12—Tb1	173.2 (7)	C2—O7—Tb2—O4	−107.2 (7)
C3—O11—Tb1—O10	29.4 (8)	C2—O7—Tb2—O3	−178.8 (8)
C3—O11—Tb1—O12	−37.7 (7)	C2—O7—Tb2—O8	141.2 (7)
C3—O11—Tb1—O14	−89.0 (9)	C4—O1—Tb2—O2	62.2 (7)
C3—O11—Tb1—O9	94.6 (7)	C4—O1—Tb2—O6	−6.4 (8)
C3—O11—Tb1—O15	174.7 (8)	C4—O1—Tb2—O7	−65.5 (7)
C3—O11—Tb1—O16	138.0 (7)	C4—O1—Tb2—O5	112.9 (7)
C3—O11—Tb1—O13	−109.2 (8)	C4—O1—Tb2—O4	−121.4 (7)
C2—O12—Tb1—O10	−30.7 (11)	C4—O1—Tb2—O3	−146.4 (7)
C2—O12—Tb1—O11	92.0 (11)	C4—O1—Tb2—O8	138.5 (7)
C2—O12—Tb1—O14	−114.9 (11)		

### Hydrogen-bond geometry (Å, °)

**Table:**

*D*—H···*A*	*D*—H	H···*A*	*D*···*A*	*D*—H···*A*
N2—H23···O12S	0.91	2.20	2.928 (15)	136
N3—H31···O17S	0.91	2.32	3.032 (15)	136
O14—H14B···O25S	0.85 (10)	2.01 (9)	2.754 (15)	145 (6)
O5—H5A···O3S^i^	0.86 (4)	2.00 (4)	2.809 (10)	156 (3)
O4—H4B···O9S^i^	0.85 (5)	2.37 (6)	3.048 (20)	137 (3)
N2—H22···O4S^ii^	0.91	2.23	3.022 (12)	145
N2—H21···O15S^ii^	0.91	2.11	2.768 (19)	129
N1—H13···O16S^ii^	0.91	2.02	2.906 (13)	163
N2—H22···O2S^ii^	0.91	2.22	3.016 (12)	147
N4—H42···O7SB^iii^	0.91	2.10	2.979 (51)	163
N3—H33···O22S^iii^	0.91	1.94	2.822 (19)	164
N4—H41···O24S^iii^	0.91	2.18	3.033 (12)	156
N4—H42···O5S^iii^	0.91	2.31	3.049 (13)	139
N1—H11···O6S^iv^	0.91	2.20	3.002 (15)	147
O3—H3B···O6S^iv^	0.85 (5)	2.33 (7)	3.149 (15)	161 (4)
N1—H12···O4S^v^	0.91	2.09	2.981 (11)	165
N2—H21···O23S^vi^	0.91	2.30	2.924 (10)	125
O3—H3A···O23S^vi^	0.85 (6)	2.04 (5)	2.882 (10)	171 (4)
O4—H4A···O20S^vi^	0.86 (6)	2.01 (4)	2.826 (10)	158 (5)
N4—H43···O19S^vii^	0.91	2.16	3.017 (11)	156
O16—H16B···O20S^vii^	0.84 (10)	2.16 (5)	2.794 (11)	133 (4)
N3—H32···O5S^viii^	0.91	2.06	2.926 (12)	159
O13—H13A···O8^ix^	0.86 (3)	2.01 (3)	2.863 (10)	174 (5)

Symmetry codes:  (i) *x*, *y*, *z*−1; (ii) *x*, *y*−1, *z*; (iii) *x*, *y*+1, *z*; (iv) *x*+1, *y*, *z*−1; (v) *x*, *y*−1, *z*−1; (vi) *x*+1, *y*, *z*; (vii) *x*, *y*, *z*+1; (viii) *x*, *y*+1, *z*−1; (ix) *x*−1, *y*, *z*.
